# Single-Step Formation of Metal Oxide Nanostructures Wrapped in Mesoporous Silica and Silica–Niobia Catalysts for the Condensation of Furfural with Acetone

**DOI:** 10.3390/nano13233046

**Published:** 2023-11-29

**Authors:** Kai Skrodczky, Margarida M. Antunes, Qingjun Zhu, Anabela A. Valente, Nicola Pinna, Patrícia A. Russo

**Affiliations:** 1 Department of Chemistry, Integrative Research Institute for the Sciences—IRIS Adlershof & The Center for the Science of Materials Berlin, Humboldt-Universität zu Berlin, Brook-Taylor-Str. 2, 12489 Berlin, Germany; kai.skrodczky@chemie.hu-berlin.de (K.S.); qingjun.zhu@hu-berlin.de (Q.Z.); 2 CICECO—Aveiro Institute of Materials, Department of Chemistry, University of Aveiro, 3810-193 Aveiro, Portugal; margarida.antunes@ua.pt (M.M.A.); atav@ua.pt (A.A.V.); 3 Deutsches Elektronen-Synchrotron DESY, Platanenallee 6, 15738 Zeuthen, Germany

**Keywords:** metal oxides, silica, nanocatalysts, biomass, furfural

## Abstract

The integration of metal oxide nanomaterials with mesoporous silica is a promising approach to exploiting the advantages of both types of materials. Traditional synthesis methods typically require multiple steps. This work instead presents a fast, one-step, template-free method for the synthesis of metal oxides homogeneously dispersed within mesoporous silica, including oxides of W, Ti, Nb, Ta, Sn, and Mo. These composites have tunable metal oxide contents, large surface areas, and wide mesopores. The combination of Nb_2_O_5_ nanoparticles (NPs) with SiO_2_ results in an increased surface area and a larger number of acid sites compared to pure Nb_2_O_5_ NPs. The surface texture and acidity of the silica–niobia composites can be tuned by adjusting the Nb/Si molar ratio. Moreover, the silica provides protection to the niobia NPs, preventing sintering during thermal treatment at 400 °C. The silica–niobia materials exhibit superior performance as catalysts in the aldol condensation of furfural (Fur) with acetone compared to pure niobia, leading to an up to 62% in product yield. Additionally, these catalysts show remarkable stability, retaining their performance over multiple runs. This work demonstrates the potential of the proposed synthesis approach for preparing more sustainable, high-performance, durable, and stable nanoscale metal oxide-based catalysts with a tunable composition, surface area, and active site density.

## 1. Introduction

Metal oxides are a versatile class of materials with applications in a variety of fields, including catalysis [1]. As catalysts or catalyst supports, they help promote a wide range of industrially relevant chemical reactions [2,3]. Nanosizing metal oxides can significantly improve catalytic efficiency due to the higher surface-to-volume ratio, which can lead to an increased number of exposed active sites and the consequent enhancement of reaction rates. Moreover, changes in the surface structure as the size is decreased to the nanoscale, such as the formation of defects, can amplify or unveil new properties. The possibility to engineer the surface structure can allow for the tuning of specific properties such as selectivity [4,5,6]. However, nanocatalysts may be difficult to separate and recover due to their small size, especially from liquid-phase reaction mixtures [7]. Catalyst regeneration also becomes challenging if oxidizing thermal treatments are required to remove organic matter from the surface, as the particles invariably coalesce and grow, which tends to diminish catalyst efficiency [8].

A strategy often employed to increase the effective surface area and stability of a catalyst is the dispersion of a nanosized material onto a silica support [2,9,10,11]. The preparation of supported catalysts normally involves the impregnation of a pre-formed silica with a metal precursor, followed by calcination under oxidizing conditions to convert the precursor into a metal oxide. This approach optimizes the use of the catalyst, enhances catalytic efficiency, facilitates catalyst recovery, and improves its thermal stability, although it cannot completely hinder particle detachment from the support’s surface during the reaction and particle growth during thermal treatments [12]. The encapsulation of nanocatalysts in mesoporous silica also increases the surface area. In addition, the surface chemistry and texture of the silica can be tailored to specific catalytic applications. The silica surrounding the catalyst nanoparticles prevents their leaching during processes taking place in the liquid phase. The high thermal stability of silica offers protection to the metal oxide nanoparticles during high-temperature regeneration processes, acting as a buffer against particle coalescence and growth [13,14,15,16]. The preparation of this type of material normally involves multiple steps, with the catalyst and silica fabricated separately or at different stages of the synthesis process. The most common methods for synthesizing mesoporous silica are aqueous sol–gel processes in the presence of templates. This may require surface functionalization of the nanoparticles to make them compatible with the solvent.

Here, we report a general, simple, and fast procedure for preparing nanosized metal oxides coated with highly porous silica. The synthesis uses non-aqueous sol–gel chemistry to produce both the metal oxide nanoparticles and mesoporous silica in one single step, from metal and silicon precursors and acetophenone as a solvent, without the use of templates. Organic solvents are well established media for synthesizing metal oxide nanomaterials that provide good control over the precursors’ reaction kinetics and therefore over the crystallinity, facilitating the fabrication of highly crystalline oxides under mild temperature conditions [17,18]. Ketones are among the solvents suitable for the non-aqueous synthesis of metal oxides, although they have been much less used than other organic solvents such as alcohols and amines [17,18,19,20]. Previously, our research has shown that acetophenone is an effective organic solvent for the synthesis of metal oxide acid nanocatalysts [19]. This solvent leads to the production of highly hydroxylated materials and promotes the development of Lewis acidity. Non-aqueous sol–gel routes to silica are more challenging and thus rarer, due to the lower reactivity of the silicon precursors compared with that of metal precursors [18]. Nevertheless, porous metallosilicates and mixed oxides have been synthesized via the reaction of metal chloride precursors with ethers (typically diisopropyl ether) or alkoxides, followed by calcination to remove the high amount of organic content (up to 20 wt. %) inherent to those synthesis approaches [18,21,22,23,24].

In this work, we synthesized high-surface-area materials consisting of oxide nanoparticles of W, Ti, Nb, Ta, Mo, or Sn, coated with mesoporous silica. The silica–niobia materials were selected for further studying the variation in the surface properties (texture and acid sites) as a function of the metal oxide content. These materials were selected, in part, due to the potential of Nb-based catalysts to exhibit water tolerance, an important characteristic for biomass-related conversion processes like Fur condensation, which yield water as a coproduct [19]. The suitability of the materials to function as catalysts was investigated in the aldol condensation reaction of furfural with acetone. To the best of our knowledge, this is the first study on the synthesis of silica-based materials using acetophenone, and the first report of Si,Nb oxide catalysts being utilized for furfural–acetone condensation. Furfural has been one of the most important bio-based industrial platform chemicals for over a century [25], and is produced via hydrolysis and dehydration reactions of hemicelluloses under acidic conditions. Furfural and its derivatives have broad applications as bio-based plastics, pharmaceuticals, agrochemicals, food/beverages, cosmetics, and solvents [26,27], but they typically possess up to five carbon atoms, which restricts their use as drop-in fuels [28]. Hence, reaction routes that increase the amount of carbon atoms, such as C-C coupling via aldol condensation, are very important [29]. The catalytic condensation of furfural with ketones gives useful intermediates to fuel-grade alkanes [29,30,31]. Fur/acetone condensation gives 4-(furan-2-yl)but-3-en-2-one (C8) and 1,5-di(furan-2-yl)penta-1,4-dien-3-one (C13), which possess comparable features to diesel fuels, i.e., they are compatible drop-in-fuels that avoid engine adjustments, etc. [31]. Additionally, C13 is an attractive intermediate to jet fuel-range hydrocarbons [32]. Silica–niobia with Nb/Si molar ratios between 0.2 and 0.82 have larger surface areas and higher amounts of acid sites than pure niobium oxide, which results in higher furfural conversions and product yields.

## 2. Materials and Methods

Chemicals: Acetophenone (99.9%), tetraethyl orthosilicate (TEOS, ≥99%), titanium(IV) isopropoxide (97%), silicon tetrachloride (99%), tungsten(VI) chloride (≥99.9%), molybdenum(VI) dichloride dioxide, and chloroform-d (CDCl_3_, 99.8 atom% D) were purchased from Sigma-Aldrich (St. Louis, MO, USA), tantalum(V) chloride (99.8%) from Alfa Aesar (Haverhill, MA, USA), and niobium(V) chloride (99.95%) from ABCR (Karlsruhe, Germany).

Synthesis: The syntheses of the silica–metal oxide materials were performed as follows. A total of 20 mL of acetophenone, 0.447 mL of TEOS, and a suitable amount of the metal precursor to make the desired molar percentage of the metal were added to a 30 mL glass microwave vial under argon. In the case of pure silica, 0.1 mL of SiCl_4_ was added to the synthesis. The vial was closed and heated in an Anton Paar Monowave 400 microwave reactor (Graz, Austria) at 220 °C for 20 min, with a heating rate of 20 °C min^−1^. The products were collected via centrifugation, washed 4 times with 20 mL of ethanol and acetone, and dried in air at 65 °C for 12 h. The SiNbx materials were tested as catalysts in the condensation of furfural with acetone. Catalyst recovery was achieved via heat treatment at 400 °C (minimal temperature for removing the organic matter from the catalyst surface), and thus, these materials were treated in the same conditions after synthesis: 400 °C for 5 h in air (heating rate of 1 °C min^−1^).

Characterization: X-ray powder diffraction (XRD) patterns were acquired using a STOE STADI MP diffractometer equipped with a MYTHEN 1K detector and a Ge monochromator (STOE & Cie GmbH, Darmstadt, Germany), in transmission configuration, using Mo Kα1 radiation (λ = 0.07093 nm). The patterns were measured in the 3–42° 2θ range, with a step size of 0.3 and time per step of 1 s. Transmission electron microscopy (TEM) and high-resolution TEM (HRTEM) micrographs were measured using an FEI Talos F200S scanning/transmission electron microscope (S/TEM) (Thermo Fisher Scientific, Waltham, MA, USA) operated at 200 kV. Nitrogen adsorption isotherms at −196 °C were measured using a Micromeritics ASAP 2020 (Norcross, GA, USA). Prior to the measurements, the samples were degassed under vacuum at 200 °C for 15 h. The mesopore volumes were determined via the t-plot method, using a macroporous silica as reference [33]. Fourier transform infrared spectroscopy (FT-IR) measurements of adsorbed pyridine were performed in a home-made transmission cell containing KBr windows and connected to a high-vacuum installation. The samples were pressed into self-supporting pellets of 13 mm diameter, degassed at 200 °C for 1 h under vacuum ≤ 5 × 10^−6^ mbar, and the spectrum was recorded. The sample was then placed in contact with 3 mbar of pyridine for 10 min and subsequently degassed at room temperature, 100, 200, and 300 °C for 1 h under high-vacuum conditions (≤5 × 10^−6^ mbar). A spectrum was recorded after each degassing treatment. The spectra were acquired using a Thermo Fischer Scientific Nicolet iS50 spectrometer (Waltham, MA, USA) equipped with an MCT detector in the range of 1700–1400 cm^−1^ with a 4 cm^−1^ resolution. The numbers of Lewis and Brønsted sites were calculated from the bands at 1455 and 1540 cm^−1^ of the differential spectra, using integrated molar extinction coefficients of 2.22 cm μmol^−1^ and 1.67 cm μmol^−1^, respectively [34]. Nuclear magnetic resonance (NMR) spectra of the reaction solution were recorded using a Bruker Avance 500 spectrometer (Billerica, MA, USA) operating at 500.13 MHz (^1^H). The solution was collected directly after reaction and subsequent centrifugation for particle separation, and mixed with CDCl_3_ for the analysis.

Catalytic tests: The catalytic reactions were carried out using tubular borosilicate batch reactors, equipped with a PTFE valve for purging and a PTFE-coated magnetic stirring bar (800 rpm). The reactor was loaded with a solution of 1.6 M Fur in acetone and 30 g_cat_ L^−1^ (mass ratio of catalyst/Fur = 0.20), and then, immersed in an oil bath heated to 140 °C. The reaction was stopped by cooling the reactors to room temperature. All substrates and products were analyzed via gas chromatography (GC), except for Fur, which was analyzed via liquid chromatography (HPLC). The GC analyses were carried out using a Thermo Scientific Trace 1300 Series GC equipped with an Agilent Technologies, Inc. (Santa Clara, CA, USA) capillary column (DB-5, 30 m × 0.32 mm × 0.25 μm; He as carrier gas) and a flame ionization detector. The HPLC analyses were carried out using a Knauer Smartline HPLC Pump 100 (Berlin, Germany) and a Shodex SH1011 H^+^ 300 mm × 8 mm (i.d.) ion exchange column (Tokyo, Japan), coupled with a Knauer Smartline UV detector 2520 (254 nm) (Berlin, Germany). The mobile phase was 0.005 M aq. H_2_SO_4_ at a flow rate of 0.8 mL min^−1^, and the column temperature was 35 °C. Calibration curves with internal standards were measured for quantification of the substrates and reaction products. Individual experiments were performed for a given reaction time, and the presented results are the mean values of at least two replicates (error < 5%). The reaction products were identified using a Shimadzu QP 2010 ultra-GC-MS (Kyoto, Japan) equipped with a HT-5 GC column (25 m × 0.32 mm × 0.10 μm) and He as carrier gas.

The used solid catalysts were separated from the reaction mixture via centrifugation at 10,000 rpm, thoroughly washed with acetone, dried at 85 °C overnight, and then, calcined at 400 °C (heating rate of 1 °C min^−1^) for 5 h. The recovered catalysts were reused for up to four consecutive 5 h batch runs of the furfural–acetone reaction, at 140 °C. The fresh catalysts were additionally placed in contact with acetone for 5 h at 140 °C, under the same conditions as those used for a normal catalytic test, but without the substrate. Afterwards, the solid was separated from the liquid phase via centrifugation. The liquid phase was then filtered using a 220 nm pore size PTFE membrane. Subsequently, the substrate was added to the obtained liquid phase to give an initial furfural concentration of 1.6 M, and this solution was left to react for 5 h at 140 °C, and finally, analyzed via chromatography.

## 3. Results and Discussion

### 3.1. General Synthesis of Metal Oxide Nanostructures Wrapped in Mesoporous Silica

**Figure 1:**
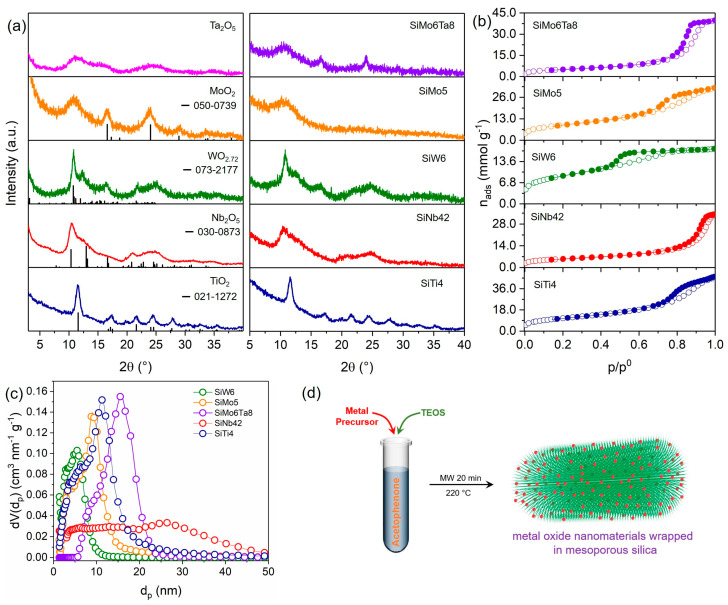
(**a**) X-ray diffractograms of pure metal oxides and SiMx, (**b**) N_2_ adsorption isotherms at −196 °C, and (**c**) pore size distributions of SiMx materials (vertical lines in (**a**) indicate reference XRD patterns of tetragonal TiO_2_ (ICDD pdf no. 021-1272), orthorhombic Nb_2_O_5_ (ICDD pdf no. 030-0873), monoclinic WO_2.72_ (ICDD pdf no. 073-2177), and hexagonal MoO_2_ (ICDD pdf no. 050-0739)). (**d**) Schematic representation of the synthesis of the SiMx materials.

The various metal oxide nanomaterials studied here could be easily synthesized through a microwave-assisted reaction of the metal precursors (e.g., alkoxides, chlorides) with acetophenone at 220 °C, via an aldol condensation mechanism. During the reaction, in addition to dypnone, which is the product of the aldol condensation of acetophenone, hydroxylated metal species are produced, which condense to form M-O-M bonds and eventually form metal oxide nanoparticles. The reaction is normally catalyzed by the Lewis acidity of the metal precursors [17,20]. For example, this synthesis produces anatase TiO_2_ NPs of 5–10 nm size from Ti(IV) isopropoxide (Figure 1a and Figure S1). The average TiO_2_ crystallite size calculated from the XRD data using the Scherrer equation is 5.9 nm. The reaction of NbCl_5_ with acetophenone results in ultrasmall (1.5–5 nm) platelet-like Nb_2_O_5_ NPs (Figure S2) with low crystallinity, as indicated by the very broad reflections in the X-ray diffraction pattern (Figure 1a) [20]. Under the same conditions, WCl_6_ leads to agglomerates of tungsten oxide nanobelts (Figure S1). The diffractogram of the material (Figure 1a) shows that a reduced oxide is formed, specifically, monoclinic WO_2.72_, (ICDD pdf no. 073-2177) which is consistent with the strong dark blue color presented by the solid. The diffractogram of MoO_2_ exhibits broad reflections above 15° (2θ) that can be indexed to hexagonal MoO_2_ (ICDD pdf no. 050-0739) and are indicative of very small crystallites. The broad reflection between 5 and 15° (2θ) also suggests the presence of amorphous molybdenum oxide [35]. The TEM images show that the material consists of layered sheets (Figure S1). Tantalum oxide derived from TaCl_5_ also has a sheet-like morphology (Figure S2) and is essentially amorphous (Figure 1a).

Tetraethylorthosilicate is much less reactive than metal precursors under similar reaction conditions and is unable to catalyze the condensation of the ketone to produce silica. However, if a metal precursor with sufficient Lewis acidity is present in the reaction mixture together with TEOS, silica and the corresponding metal oxide are produced simultaneously. This likely happens due to the stronger Lewis acidity of the metals, which can coordinate with the ethoxy groups of TEOS and decrease the electron density at the silicon atom, thus making it more susceptible to a nucleophilic attack by the carbonyl oxygen of the ketone. ^1^H NMR analysis of the post-reaction liquid mixture (Figure S3) shows the presence of dypnone and ethanol, the latter being a side-product of the reaction of TEOS with acetophenone, which indicates that the oxygen for the formation of the silica comes from the ketone. A general reaction scheme and a proposed reaction mechanism for the synthesis of the metal oxide–silica composites in acetophenone are shown in Figures S4 and S5, respectively.

This synthesis strategy was used to fabricate various metal oxide–silica materials (Figure 1d). The samples are named SiMx, where M = Ti, W, Sn, Nb, Mo, Ta, and x is the atomic percentage of metal in the sample with respect to Si (Table S1). Most of the metal precursors studied here are sufficiently reactive to promote the reaction. When this is not the case, a catalytic amount of SiCl_4_ can be added to the synthesis mixture to initiate the condensation. Pure silica (SiO_2_) can be synthesized in this way, by reacting TEOS and SiCl_4_ with acetophenone.

The XRD patterns of several representative SiMx materials are shown in Figure 1a. The diffractograms are similar to those of the corresponding pure metal oxides in Figure 1a, suggesting that the materials are made of a combination of metal oxide particles and silica. Therefore, the peaks in the SiTi4 diffractogram are all indexed to the anatase phase of TiO_2_ (ICDD pdf no. 021-1272) The patterns of SiW6 and SiNb42 are identical to those of the respective pure metal oxides, showing the presence of WO_2_._72_ and Nb_2_O_5_ NPs, respectively, in their composition. All the SiMx samples containing Mo have black color, which is consistent with the presence of MoO_2_. The diffractogram of SiMo6Ta8 shows two broad peaks at 16.6 and 24.0° ascribed to hexagonal MoO_2_, but no reflections from Ta_2_O_5_ are distinguishable due to its low crystallinity. The XRD pattern of SiMo5 only shows a very broad reflection between 5 and 15° that is typical of amorphous materials, while the peaks of hexagonal MoO_2_ are not clearly observed in the pattern, suggesting that the MoO_2_ particles are very small and/or of low crystallinity.

**Figure 2:**
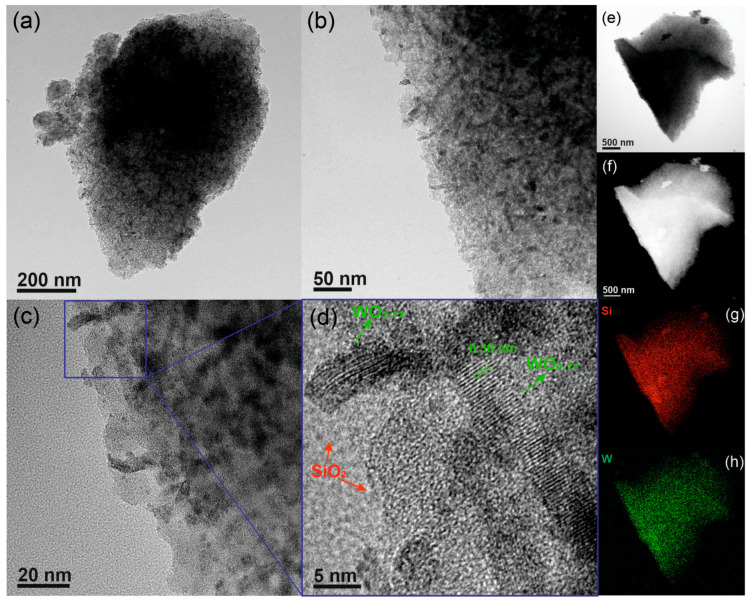
(**a**–**d**) TEM and HRTEM, (**e**) BF-, and (**f**) HAADF-STEM images, and (**g**) Si and (**h**) W elemental maps of SiW6.

The TEM images of SiW6 in Figure 2 show that the material consists of large particles made of tungsten oxide nanobelts embedded in a silica matrix (Figure 2a–c). Crystalline nanobelts with width of ca. 5 nm are seen in the HRTEM image in Figure 2d, surrounded by amorphous silica. The presence of lattice fringes indicates the tungsten oxide is well crystallized. The spacings of 0.380 nm match the interplanar distance for the (010) planes of WO_2_._72_, corresponding to the most intense reflection in the XRD pattern at 10.8° (2θ). The images, in particular, the high-angular annular dark-field scanning transmission electron microscopy (HAADF-STEM) image (Figure 2f), also suggests that the silica-WO_2_._72_ composite is highly porous. Moreover, the energy-dispersive X-ray spectroscopy (EDS) Si and W elemental maps reveal that the tungsten oxide nanobelts are uniformly dispersed in the porous silica matrix (Figure 2g,h). The microscopy imaging results are qualitatively similar for the SiMx containing other metal oxides (Figures S6 and S7), i.e., the materials consist of dispersed metal oxide nanoparticles surrounded by porous silica, which is confirmed by the elemental maps. The main differences between the samples seem to be caused by the different amounts of metal oxide in the materials, which affects their morphology. For higher metal oxide contents, the samples gradually become more of a network of interconnected silica particles with embedded metal oxide (Figures S7–S9).

The porosity of the SiMx materials was confirmed by N_2_ adsorption measurements. The N_2_ adsorption isotherms at −196 °C of all the samples are type IV (Figure 1b), which are typical of mesoporous solids. Pure silica synthetized via the same method is also mesoporous and has a high surface area of more than 1000 m^2^ g^−1^ (Table 1). The high surface areas and large pore sizes of SiMx (Table 1) contrast with the textural properties of the pure metal oxides, which have relatively small surface areas and do not exhibit intrinsic porosity. The BET surface areas of the pure metal oxides are 44, 118, 185, and 196 m^2^ g^−1^ for MoO_2_, Ta_2_O_5_, WO_2_._72_, and Nb_2_O_5_, respectively. The surface area of the SiMx materials is influenced by both the type and quantity of metal oxide in the sample (Table 1 and Table 2). Typically, an increase in metal oxide content leads to a reduction in surface area, which is likely also affected by the morphology of the metal oxide particles. While the pore size also varies, establishing a correlation between it and the metal oxide content is not possible. Nevertheless, the areas still remain very high compared to the pure oxide, even for SiMx with metal contents such as 42 at. %. All the SiMx samples have large mesopores, which can provide easy accessibility to the surface of the metal oxide particles, although the size of the pores varies with the type of metal and metal amount. The pore size distribution (PSD) width also depends on the sample, on the type of metal, and especially on the amount of metal (Figure 1c), and tends to broaden as the metal amount increases. Nevertheless, typically, the PSDs are relatively narrow considering that no type of template is used in the synthesis, although the acetophenone and dypnone molecules present in the reaction mixture may partially act as surfactants. Despite the pore size being less controllable compared to traditional synthesis methods that use surfactants, the absence of surfactants eliminates the need for extra synthetic steps for surfactant removal, making the process greener. The combination of the porous silica with the metal oxide particles has clear benefits in terms of the textural properties of the metal oxides, resulting in the increase in the surface area. This synthesis procedure allows us to take advantage of the organic solvent to produce nanocrystalline metal oxides, while combining it with a highly porous silica that provides accessibility through the large mesopores.

### 3.2. Surface and Catalytic Properties of Silica–Niobia Structures

The silica–niobia materials (SiNbx) were selected for carrying out a more detailed study on the effect of the metal oxide content on the textural, acidic, and catalytic properties. The samples investigated had Nb contents of 7, 20, 42, 65, and 82 at. % with respect to Si, and were subjected to thermal treatment at 400 °C in air, which is the regeneration treatment of the catalyst. TEM imaging shows that while the catalyst regeneration treatment of the pure Nb_2_O_5_ NPs leads to sintering, the coalescence of nanoparticles, and an increase in particle size (Figure S10), no significant changes are observed for the SiNbx materials, demonstrating the protective effect of the silica against sintering. Therefore, the crystallinity degree and size of the metal oxide NPs in SiNbx are maintained, despite the use of thermal regeneration treatments.

**Figure 3:**
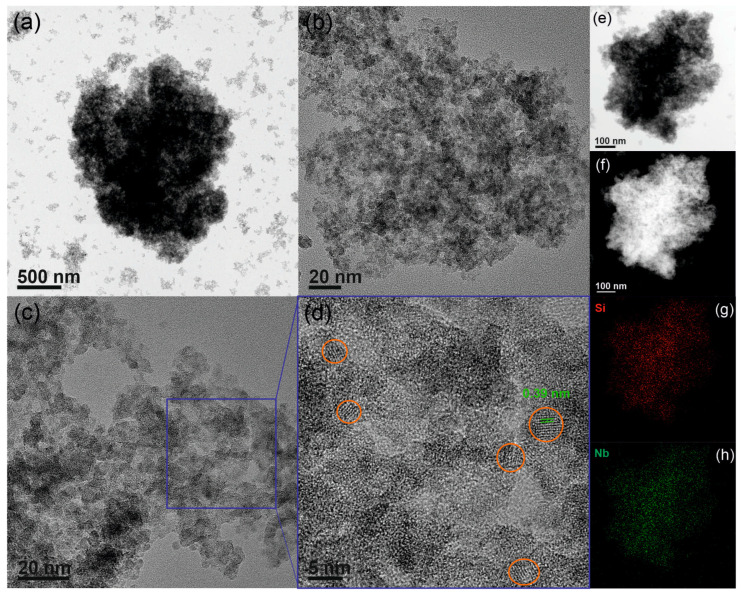
(**a**–**d**) TEM and HRTEM, (**e**) BF-, and (**f**) HAADF-STEM images, and (**g**) Si and (**h**) Nb elemental maps of SiNb42.

The XRD patterns of all the SiNbx materials exhibit similarly broad reflections, indicative of small crystallites and low crystallinity, regardless of the amount of niobium oxide incorporated into the sample (Figure S11). Figure 3 shows TEM images of SiNb42. The overview images show that the material consists of silica–niobia particles of different sizes. Magnification of the square area indicated in Figure 3c shows small relative crystalline nanoparticles with sizes of around 5 nm (indicated by orange circles) surrounded by regions of amorphous silica. Pores are also observed on the images as well as in the HAADF-STEM image (Figure 3f), where dark regions, especially noticeable on the edges of the particle, correspond to pores. The interplanar distance determined from the HRTEM data (0.39 nm) is consistent with the d spacing of the (001) planes of the orthorhombic structure of Nb_2_O_5_. The Si and Nb elementary maps demonstrate that the niobium oxide particles are well dispersed among the silica. The morphology of the silica–niobia materials varies with increasing niobium content. For Nb < 42 at. %, the samples are best described as Nb_2_O_5_ NPs dispersed in a porous silica matrix (Figure S9), and for Nb ≥ 42 at. %, the samples are best described as interconnected nanoparticles made of Nb oxide and silica (Figure 3 and Figure S10). Nevertheless, the size of the Nb_2_O_5_ particles and their homogeneous dispersion within the silica is maintained, regardless of the Nb amount on the sample.

The surface areas and mesopore volumes of the SiNbx solids decrease with increasing Nb_2_O_5_ in the sample, although even for SiNb82, the surface area is higher than that of the pure metal oxide. All the silica–niobia samples have large mesopores, whose size increases with increasing Nb content up to x = 82, and then, decreases again (Table 2, Figure S11).

**Figure 4:**
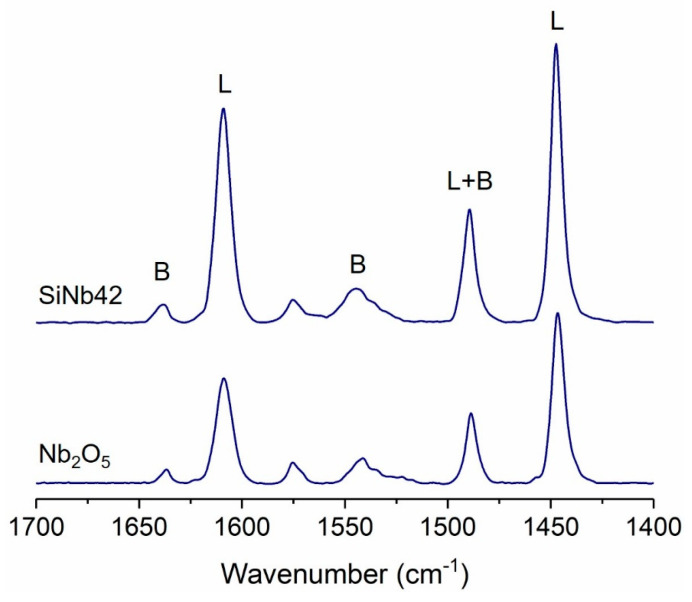
FT-IR spectra of pyridine adsorbed on SiNb42 and Nb_2_O_5_ at 100 °C.

FT-IR spectroscopy of adsorbed pyridine was used to evaluate the acid properties of the solids. Both SiNbx materials and Nb_2_O_5_ NPs have Lewis and Brønsted acid sites, as indicated by the typical bands of pyridine adsorbed on Lewis and Brønsted acid sites in the spectra of Figure 4 [36,37], with a higher proportion of the former (Table 2). Moreover, the sites are relatively weak, especially the Brønsted sites, from which pyridine is almost completely desorbed at 200 °C. In the case of the Lewis sites, almost complete desorption occurs at 300 °C (Figure S12). The number of acid sites (AS) increases with increasing Nb content, reaching maximum values for SiNb42, followed by SiNb65. The number of Lewis AS and Brønsted AS follows the same trend, although the number of Lewis acid sites varies more. The quantity of active sites is determined by the balance between the amount of metal oxide in the sample, which provides the acid sites, and the dispersion of the metal in the porous silica matrix that is achieved, and is associated with the surface area of the material. The combination of the metal oxide with the silica affects the Lewis-to-Brønsted ratio (L/B), which increases with increasing Nb content. For example, it is 1.8 for SiNb7, 2.9 for SiNb42, and 4.0 for Nb_2_O_5_ NPs. Additionally, this combination leads to the optimization of metal usage. Therefore, for example, the number of acid sites per mmol of Nb present in SiNb42 is 57.6 μmol_AS_ mmol_Nb_^−1^, while it is 19.9 μmol_AS_ mmol_Nb_^−1^ for Nb_2_O_5_. These results show that the silica–niobia offers some advantages compared to the pure Nb_2_O_5_: it allows for thermal regeneration of the catalysts without changes in the crystallinity and particle size, as well as obtaining more acidic materials with significantly less metal.

The SiNbx materials were investigated as catalysts for the aldol condensation reaction of furfural with acetone at 140 °C. The main reaction products were 4-(furan-2-yl)but-3-en-2-one, 6-(furan-2-yl)-4-methylhexa-3,5-dien-2-one (C11), 1,5-di(furan-2-yl)penta-1,4-dien-3-one, and 4-(furan-2-yl)-5-(furan-2-ylmethyl)hept-3-ene-2,6-dione (C16). The predominant reaction product was C8. The catalytic results are discussed considering the C8 yield and the total product yield (C8–C16), since they may be, for example, useful fuel blends. While the Fur-acetone reaction system without a catalyst was sluggish (5% conversion at 140 °C/5 h, and no significant formation of C8–C16 products), the SiNbx materials led to 53–75% conversions at 5 h of reaction, with mainly C8 formation (39–62% yield).

**Figure 5:**
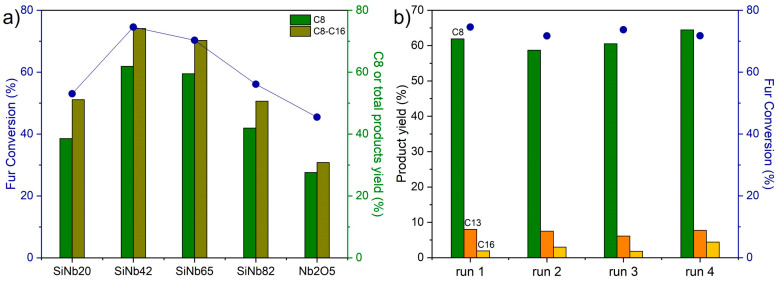
(**a**) Influence of the Nb oxide content of the SiNbx materials on furfural conversion, C8 yield, and total C8–C16 product yield; (**b**) consecutive catalytic runs of the furfural–acetone condensation reaction over SiNb42 (C8OH and C11 yields were always less than 2%). Reaction conditions: 1.6 M Fur in acetone, 140 °C, 5 h, 30 g_cat_ L^−1^.

The Nb content of the SiNbx materials influences catalytic performance (Figure 5a). The Fur conversion and product yields increase with increasing Nb content up to x = 42. The performance of SiNb65 is comparable to that of SiNb42; however, a further increase in the Nb amount from 65 to 82 does not have a beneficial effect on the catalytic reaction (Figure 5a). All the silica–niobia compounds, regardless of the metal content, outperform the pure Nb_2_O_5_ catalyst, which leads to relatively low product yields (28% of C8 yield and 31% of C8–C16 yield), demonstrating benefits to the catalytic performance of the combination of niobia nanoparticles with silica.

The highest conversions, C8 yield, and C8–C16 yield are obtained with SiNb42, followed closely by SiNb65, which are the catalysts that have the highest number of total acid sites. Therefore, the catalytic performance correlates with the acidity of the samples, which, in turn, is determined by the balance between the amount of Nb oxide incorporated into the sample and the surface area of the final product. This allows for more efficient utilization of the catalytically active material. Similarly, the initial activities are at maximum for the SiNb42 and SiNb65 catalysts (ca. 20 mmol g_cat_^−1^ h^−1^), and decrease to ca. 15 mmol g_cat_^−1^ h^−1^ for SiNb82 and to ca. 10 mmol g_cat_^−1^ h^−1^ for SiNb20. The L/B ratio also seems to play a role in catalytic performance. SiNb20 has a higher number of total (195 µmol g^−1^) and Lewis acid sites (137 µmol g^−1^) than SiNb82 (156 and 123 µmol g^−1^, respectively). In contrast, the C8 yield and initial activity are higher for the latter, which could be associated with its higher L/B ratio (3.7) compared to that of SiNb20 (2.4). Moreover, although C16 is formed in small amounts, its highest yield was obtained for SiNb20 (ca. 5% compared with 1–2% for the remaining catalysts), which could be due to the lower L/B ratio of SiNb20 compared to the other materials [38,39]. Overall, these results suggest that high Nb content combined with high S_BET_ is desirable, although the synthesis methodology led to a maximum of S_BET_ and acid site concentration for intermediate Nb content (x = 42).

Catalytic stability was studied by performing several consecutive catalytic batch runs. The solids showed a brownish coloration after the first catalytic run, due to the presence of organic matter at the surface (e.g., sample SiNb42 contained 27 wt. % of carbon after catalysis), which is consistent with the literature on coke formation during the furfural–acetone condensation over solid acid catalysts [40]. The catalyst can be completely regenerated via calcination at 400 °C in air. Figure 5b shows the furfural conversion and product yields obtained for SiNb42 over four consecutive catalytic runs and regeneration processes. The catalytic performance is relatively steady over the four runs, and the product distributions remain similar, indicating that the silica–niobia material is stable with respect to thermal regeneration, which allows it to be reused without loss of activity. The stability of the catalyst is further confirmed via characterization of the recovered and regenerated material, which shows no significant differences in terms of composition, structure, morphology, the size of the metal oxide nanoparticles, or textural properties, as the XRD pattern, N_2_ adsorption isotherm, surface area (440 m^2^ g^−1^), and TEM images of the recovered SiNb42 catalyst are similar to the initial ones (Figures S13 and S14). The stability of the catalyst against leaching of the active material was also investigated. First, the catalyst was contacted with acetone for 5 h at 140 °C in the absence of a substrate. After separating the solid via centrifugation, furfural was added to the liquid to give an initial concentration of 1.6 M, and reacted for 5 h at 140 °C. This resulted in furfural conversion of 5%, which is similar to that of the blank (i.e., without a catalyst), suggesting that no Nb_2_O_5_ or other Nb species was leached from the samples under catalytic reaction conditions.

To the best of our knowledge, this is the first study to report on Si,Nb oxide catalysts for the targeted reaction. Table S2 compares the performance of SiNb42 with that of various fully inorganic silica-based catalysts previously reported in the literature for the Fur/acetone condensation reaction. Zirconium (25 wt. %) on mesoporous silica [41] and a copper-loaded mesoporous Cu/Al-MCM-41 material [42] showed relatively good results based on C8 yields. However, the zirconium catalyst was used in more than double the weight percentage of the catalyst relative to Fur, and the Cu/Al-MCM-41 was tested at a higher temperature and for a longer reaction time. Additionally, both reported catalysts used a larger amount of acetone, especially in the case of Cu/Al-MCM-41, compared to that used for SiNb42. In contrast, magnesium-containing base catalysts were tested at a lower temperature (50 °C) but resulted in significantly lower C8 yields [43,44].

## 4. Conclusions

A series of SiMx nanocomposites (M = Ti, W, Nb, Sn, Ta, Mo), consisting of nanosized metal oxides uniformly distributed within a mesoporous silica matrix, were successfully synthesized via a one-step microwave-assisted procedure without the use of templates. These composites have tunable compositions, and high surface areas and mesopore volumes, compared to the pure metal oxides synthesized in the same way. More detailed investigations on SiNbx demonstrated the protective effect of silica against the sintering of metal oxide NPs during thermal treatments. The surface area, number of Brønsted and Lewis acid sites, and B/L ratio could be tuned by controlling the Nb_2_O_5_ content, allowing for the preparation of solids with higher quantities of acid sites than pure Nb_2_O_5_. SiNbx were employed as heterogenous catalysts in the aldol condensation of furfural with acetone at 140 °C, outperforming the Nb_2_O_5_ NPs, and showing high recyclability. Their catalytic activity correlated with their acidity. This work shows the potential of this synthesis approach to develop diverse heterogeneous catalysts based on metal oxide nanomaterials with diverse compositions and properties.

## Figures and Tables

**Table 1:** Textural properties of the SiMx materials.

Sample	S_BET_ (m^2^ g^−1^) ^1^	V_mp_ (cm^3^ g^−1^) ^2^	dp (nm) ^3^
SiO_2_	1196	1.40	9.1
SiTi4	895	1.27	11.3
SiW6	724	0.58	5.5
SiMo5	755	0.94	9.1
SiMo6Ta8	413	1.26	15.5

^1^ BET specific surface area; ^2^ mesopore volume determined by the t-plot; ^3^ pore size (maximum of the DFT pore size distribution).

**Table 2:** Textural and acid properties of the SiNbx materials.

Sample	S_BET_ (m^2^ g^−1^) ^1^	V_mp_ (cm^3^ g^−1^) ^2^	dp (nm) ^3^	AS_T_ (μmol g^−1^) ^4^	AS_L_ (μmol g^−1^) ^5^	AS_B_ (μmol g^−1^) ^6^
SiNb7	913	1.02	7.3	138	88	50
SiNb20	682	1.40	18.6	195	137	58
SiNb42	449	0.79	26.4	267	198	69
SiNb65	296	0.42	27.2	252	190	62
SiNb82	224	0.25	17.3	156	123	33
Nb_2_O_5_	161	-	-	150	120	30

^1^ BET specific surface area; ^2^ mesopore volume determined by the t-plot; ^3^ pore size (maximum of the DFT pore size distribution); ^4^ total number of acid sites; ^5^ number of Lewis acid sites; ^6^ number of Brønsted acid sites.

## Data Availability

The data presented in this study are available on request from the corresponding author.

## Supplementary Materials

The following supporting information can be downloaded at: https://www.mdpi.com/article/10.3390/nano13233046/s1, Figure S1: TEM images of (**a**,**b**) TiO_2_, (**c**,**d**) WO_2.72_, and (**c**,**d**) MoO_2_; Figure S2: TEM images of (**a**,**b**) Nb_2_O_5_ and (**c**,**d**) Ta_2_O_5_; Figure S3: ^1^H-NMR spectrum of the reaction solution of the reaction of NbCl_5_ and TEOS with acetophenone; Figure S4: General reaction scheme of the synthesis of metal oxide–silica composites in acetophenone; Figure S5: Proposed general reaction mechanism for the hydroxylation of the metal oxide precursor (chloride, alkoxide) via aldol condensation of acetophenone and formation of dypnone; Table S1: Composition of the mesoporous silica-metal oxide materials; Figure S6: TEM and HAADF-STEM images of (**a**–**c**) SiO_2_, (**d**–**g**) SiTi4, and (**h**–**k**) SiSn5, and corresponding elemental maps; Figure S7: TEM and HAADF-STEM images of (**a**–**d**) SiMo5 and (**e**–**h**) SiMo6Ta8, and corresponding elemental maps; Figure S8: TEM and HAADF-STEM images of (**a**–**d**) SiNb7 and (**e**–**g**) SiNb20, and corresponding elemental maps; Figure S9: TEM and HAADF-STEM images of (**a**–**c**) SiNb65 and (**d**–**g**) SiNb82, and corresponding elemental maps; Figure S10: TEM images of Nb_2_O_5_ NPs calcined at 400 °C in air; Figure S11: (**a**) XRD patterns and (**b**) N_2_ adsorption isotherms at −196 °C of SiNbx materials; Figure S12: FT-IR spectra of pyridine adsorbed on SiNb42 at 100 °C, 200 °C, and 300 °C; Figure S13: (**a**) XRD pattern and (**c**) N_2_ adsorption isotherm at −196 °C of SiNb42 after catalytic tests and thermal regeneration treatments at 400 °C in air; Figure S14: TEM images of SiNb42 after catalysis and thermal treatments at 400 °C in air; Table S2: Catalytic results for SiNb42 and literature data for fully inorganic silica-based catalysts, tested for Fur/acetone condensation.

## References

[1] Chavali M.S., Nikolova M.P. (2019). Metal oxide nanoparticles and their applications in nanotechnology. SN Appl. Sci..

[2] Lee D.W., Yoo B.R. (2014). Advanced metal oxide (supported) catalysts: Synthesis and applications. J. Ind. Eng. Chem..

[3] Védrine J.C. (2019). Importance, features and uses of metal oxide catalysts in heterogeneous catalysis. Chin. J. Catal..

[4] Xie C., Yan D., Li H., Du S., Chen W., Wang Y., Zou Y., Chen R., Wang S. (2020). Defect Chemistry in Heterogeneous Catalysis: Recognition, Understanding, and Utilization. ACS Catal..

[5] Wang G., Yang Y., Han D., Li Y. (2017). Oxygen defective metal oxides for energy conversion and storage. Nano Today.

[6] Jia J., Qian C., Dong Y., Li Y.F., Wang H., Ghoussoub M., Butler K.T., Walsh A., Ozin G.A. (2017). Heterogeneous catalytic hydrogenation of CO_2_ by metal oxides: Defect engineering—Perfecting imperfection. Chem. Soc. Rev..

[7] Yan N., Xiao C., Kou Y. (2010). Transition metal nanoparticle catalysis in green solvents. Coord. Chem. Rev..

[8] Hansen T.W., DeLaRiva A.T., Challa S.R., Datye A.K. (2013). Sintering of Catalytic Nanoparticles: Particle Migration or Ostwald Ripening?. Acc. Chem. Res..

[9] Lwin S., Wachs I.E. (2014). Olefin Metathesis by Supported Metal Oxide Catalysts. ACS Catal..

[10] Nava R., Pawelec B., Castaño P., Álvarez-Galván M.C., Loricera C.V., Fierro J.L.G. (2009). Upgrading of bio-liquids on different mesoporous silica-supported CoMo catalysts. Appl. Catal. B.

[11] Esposito S. (2019). “Traditional” Sol-Gel Chemistry as a Powerful Tool for the Preparation of Supported Metal and Metal Oxide Catalysts. Materials.

[12] McCarty J.G., Gusman M., Lowe D.M., Hildenbrand D.L., Lau K.N. (1999). Stability of supported metal and supported metal oxide combustion catalysts. Catal. Today.

[13] Ge J., Zhang Q., Zhang T., Yin Y. (2008). Core–Satellite Nanocomposite Catalysts Protected by a Porous Silica Shell: Controllable Reactivity, High Stability, and Magnetic Recyclability. Angew. Chem. Int. Ed..

[14] Yang H., Cui X., Li S., Cen Y., Deng T., Wang J., Olsbye U., Fan W. (2020). Developing a general method for encapsulation of metal oxide nanoparticles in mesoporous silica shell by unraveling its formation mechanism. Microporous Mesoporous Mater..

[15] Forman A.J., Park J.-N., Tang W., Hu Y.-S., Stucky G.D., McFarland E.W. (2010). Silica-Encapsulated Pd Nanoparticles as a Regenerable and Sintering-Resistant Catalyst. ChemCatChem.

[16] Fang R., Tian P., Yang X., Luque R., Li Y. (2018). Encapsulation of ultrafine metal-oxide nanoparticles within mesopores for biomass-derived catalytic applications. Chem. Sci..

[17] Deshmukh R., Niederberger M. (2017). Mechanistic Aspects in the Formation, Growth and Surface Functionalization of Metal Oxide Nanoparticles in Organic Solvents. Chem. Eur. J..

[18] Mutin P.H., Vioux A. (2013). Recent advances in the synthesis of inorganic materials via non-hydrolytic condensation and related low-temperature routes. J. Mater. Chem. A.

[19] Skrodczky K., Antunes M.M., Han X., Santangelo S., Scholz G., Valente A.A., Pinna N., Russo P.A. (2019). Niobium pentoxide nanomaterials with distorted structures as efficient acid catalysts. Commun. Chem..

[20] Beach E.R., Shqau K., Brown S.E., Rozeveld S.J., Morris P.A. (2009). Solvothermal synthesis of crystalline nickel oxide nanoparticles. Mater. Chem. Phys..

[21] Debecker D.P., Bouchmella K., Poleunis C., Eloy P., Bertrand P., Gaigneaux E.M., Mutin P.H. (2009). Design of SiO_2_−Al_2_O_3_−MoO_3_ Metathesis Catalysts by Nonhydrolytic Sol−Gel. Chem. Mater..

[22] Bouchmella K., Hubert Mutin P., Stoyanova M., Poleunis C., Eloy P., Rodemerck U., Gaigneaux E.M., Debecker D.P. (2013). Olefin metathesis with mesoporous rhenium–silicium–aluminum mixed oxides obtained via a one-step non-hydrolytic sol–gel route. J. Catal..

[23] Dochain D.D., Stýskalík A., Debecker D.P. (2019). Ag- and Cu-Promoted Mesoporous Ta-SiO_2_ Catalysts Prepared by Non-Hydrolytic Sol-Gel for the Conversion of Ethanol to Butadiene. Catalysts.

[24] Smeets V., Ben Mustapha L., Schnee J., Gaigneaux E.M., Debecker D.P. (2018). Mesoporous SiO_2_-TiO_2_ epoxidation catalysts: Tuning surface polarity to improve performance in the presence of water. Mol. Catal..

[25] Mathew A.K., Abraham A., Mallapureddy K.K., Sukumaran R.K., Bhaskar T., Pandey A., Mohan S.V., Lee D.-J., Khanal S.K. (2018). Waste Biorefinery.

[26] Mariscal R., Maireles-Torres P., Ojeda M., Sádaba I., López Granados M. (2016). Furfural: A renewable and versatile platform molecule for the synthesis of chemicals and fuels. Energy Environ. Sci..

[27] Truong C.C., Verma V.K., Mishra P., Suh Y.-W., Mishra D.K., Li H., Saravanamurugan S., Pandey A., Elumalai S. (2022). Biomass, Biofuels, Biochemicals.

[28] Wegenhart B.L., Yang L., Kwan S.C., Harris R., Kenttämaa H.I., Abu-Omar M.M. (2014). From Furfural to Fuel: Synthesis of Furoins by Organocatalysis and their Hydrodeoxygenation by Cascade Catalysis. ChemSusChem.

[29] Bohre A., Dutta S., Saha B., Abu-Omar M.M. (2015). Upgrading Furfurals to Drop-in Biofuels: An Overview. ACS Sustain. Chem. Eng..

[30] Zang H., Wang K., Zhang M., Xie R., Wang L., Chen E.Y.X. (2018). Catalytic coupling of biomass-derived aldehydes into intermediates for biofuels and materials. Catal. Sci. Technol..

[31] Huber G.W., Chheda J.N., Barrett C.J., Dumesic J.A. (2005). Production of Liquid Alkanes by Aqueous-Phase Processing of Biomass-Derived Carbohydrates. Science.

[32] Zitouni A., Bachir R., Bendedouche W., Bedrane S. (2021). Production of bio-jet fuel range hydrocarbons from catalytic HDO of biobased difurfurilydene acetone over Ni/SiO_2_-ZrO_2_ catalysts. Fuel.

[33] Jaroniec M., Kruk M., Olivier J.P. (1999). Standard Nitrogen Adsorption Data for Characterization of Nanoporous Silicas. Langmuir.

[34] Emeis C.A. (1993). Determination of Integrated Molar Extinction Coefficients for Infrared Absorption Bands of Pyridine Adsorbed on Solid Acid Catalysts. J. Catal..

[35] Koziej D., Rossell M.D., Ludi B., Hintennach A., Novák P., Grunwaldt J.-D., Niederberger M. (2011). Interplay Between Size and Crystal Structure of Molybdenum Dioxide Nanoparticles—Synthesis, Growth Mechanism, and Electrochemical Performance. Small.

[36] Busca G. (1998). Spectroscopic characterization of the acid properties of metal oxide catalysts. Catal. Today.

[37] Tamura M., Shimizu K.-i., Satsuma A. (2012). Comprehensive IR study on acid/base properties of metal oxides. Appl. Catal. A.

[38] Kikhtyanin O., Kelbichová V., Vitvarová D., Kubů M., Kubička D. (2014). Aldol condensation of furfural and acetone on zeolites. Catal. Today.

[39] Kikhtyanin O., Kubička D., Čejka J. (2015). Toward understanding of the role of Lewis acidity in aldol condensation of acetone and furfural using MOF and zeolite catalysts. Catal. Today.

[40] Kikhtyanin O., Bulánek R., Frolich K., Čejka J., Kubička D. (2016). Aldol condensation of furfural with acetone over ion-exchanged and impregnated potassium BEA zeolites. J. Mol. Catal. A.

[41] Balaga R., Yan P., Ramineni K., Du H., Xia Z., Marri M.R., Zhang Z.C. (2022). The Role and Performance of Isolated Zirconia Sites on Mesoporous Silica for Aldol Condensation of Furfural with Acetone. Appl. Catal. A Gen..

[42] Gandhi P., Saha B., Vedachalam S., Dalai A.K. (2023). Renewable Fuel Intermediates from Furfural over Copper-Loaded Mesoporous Aldol Condensation Catalysts. Sustain. Energy Fuels.

[43] Arumugam M., Kikhtyanin O., Osatiashtiani A., Kyselová V., Fila V., Paterova I., Wong K.L., Kubička D. (2023). Potassium-Modified Bifunctional MgAl-SBA-15 for Aldol Condensation of Furfural and Acetone. Sustain. Energy Fuels.

[44] Kondratowicz T., Slang S., Dubnová L., Kikhtyanin O., Belina P., Capek L. (2022). Controlled Silica Core Removal from SiO_2_@MgAl Core-Shell System as a Tool to Prepare Well-Oriented and Highly Active Catalysts. Appl. Clay Sci..

