# Supplementary material for: Single-Step Formation of Metal Oxide Nanostructures Wrapped in Mesoporous Silica and Silica–Niobia Catalysts for the Condensation of Furfural with Acetone
Source: Nanomaterials (Basel). 2023 Nov 29;13(23):3046. doi: 10.3390/nano13233046 (PMC10708440; doi:10.3390/nano13233046)
Supplement: Supplementary file 1 [file nanomaterials-13-03046-s001.zip › nanomaterials-2736054-supplementary.pdf]

Supplementary Materials

Single-Step Formation of Metal Oxide Nanostructures Wrapped in Mesoporous Silica and Silica-Niobia Catalysts for the Condensation of Furfural with Acetone

Kai Skrodczky* [1]*, Margarida M. Antunes* [2]*, Qingjun Zhu* [1,3]*, Anabela A. Valente* [2]*, Nicola Pinna* [1,]* *and Patrícia A. Russo* [1,]

[1] *Department of Chemistry, IRIS Adlershof & The Center for the Science of Materials Berlin, Humboldt-Universität zu Berlin, Brook-Taylor-Str. 2, 12489 Berlin, Germany*

[2] *Department of Chemistry, CICECO, University of Aveiro, Aveiro, Portugal*

[3] *Deutsches Elektronen-Synchrotron DESY, Platanenallee 6, 15738 Zeuthen, Germany*

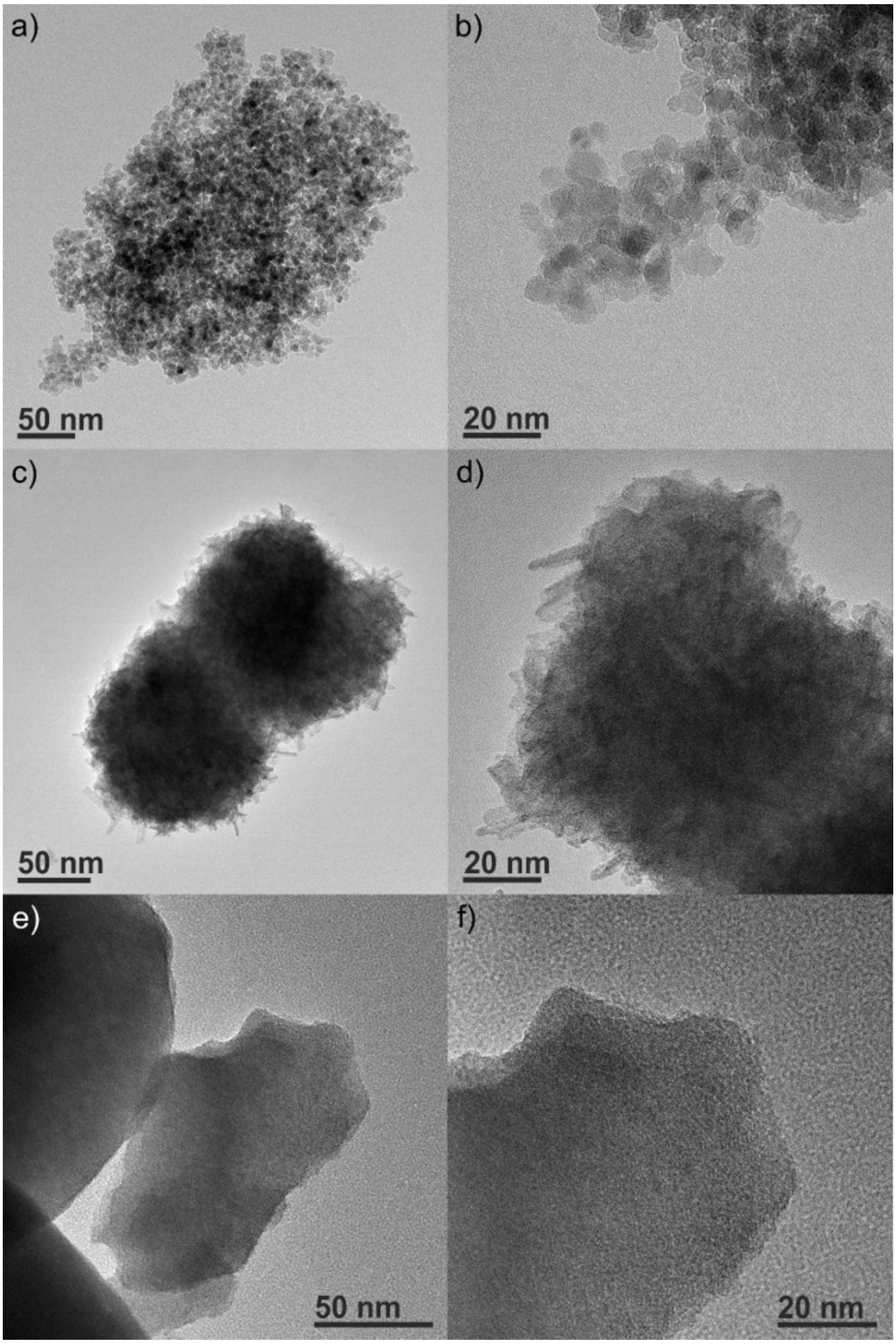

Figure S1. TEM images of a,b) TiO_2, c,d) $WO_{2.72}$, and c,d) MoO_2.

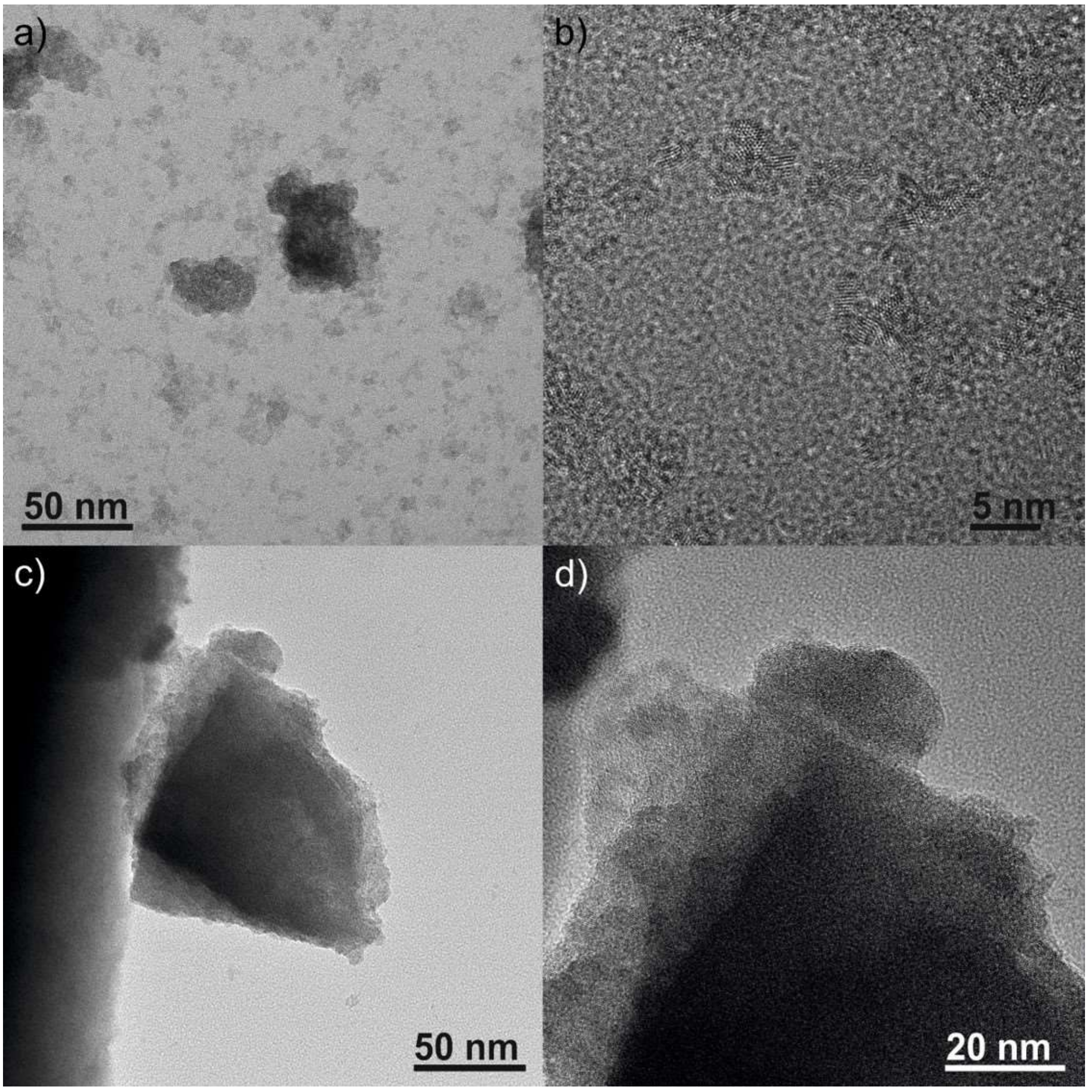

Figure S2. TEM images of a,b) Nb_2O_5 and c,d) Ta_2O_5.

NMR

^{1}H-NMR spectroscopy of the reaction liquid after particle separation (Figure S3) showed unreacted acetophenone as the main component (^{1}H-NMR (500 MHz, $CDCl_3$) δ = 7.95 – 7.93 (m, $2H_{arom.}$), 7.57 – 7.52 (m, $1H_{arom.}$), 7.47 – 7.39 (m, $2H_{arom.}$), 2.57 (s, $3H_{methyl}$) ppm).
Furthermore, there can be found additional signals which can be assigned to dypnone, the product of acetophenone aldol-condensation at δ = 8.00 – 7.98 (m, $2H_{arom.}$), 7.16 (q, $1H_{allylic}$) and 2.63 (d, $3H_{methyl}$) ppm. The other signals for the aromatic protons of dypnone might be overlapped by the signals of acetophenone in this region.
Additionally, there are traces of TEOS and ethanol visible (δ = 3.71 (q, 2H), 1.23 (t, 3H) and δ = 3.52 (q, 2H), 1.45 (t, 3H), respectively) indicating incomplete condensation of TEOS to silica.

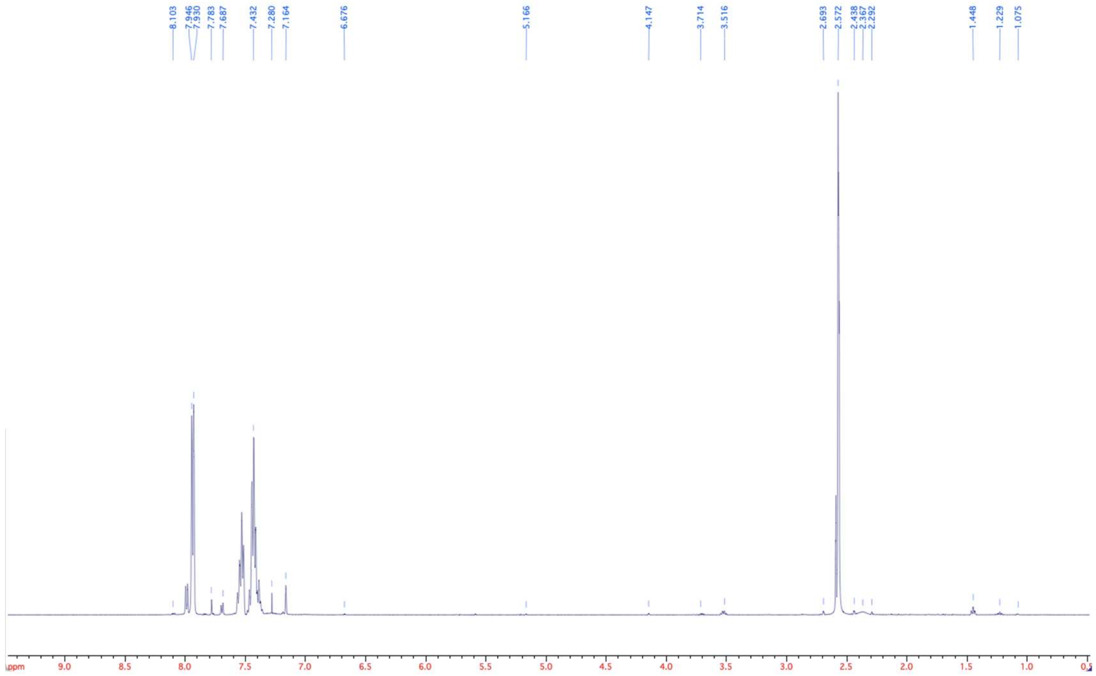

Figure S3. ^{1}H-NMR spectrum of the reaction solution of the reaction of $NbCl_5$ and TEOS with acetophenone.

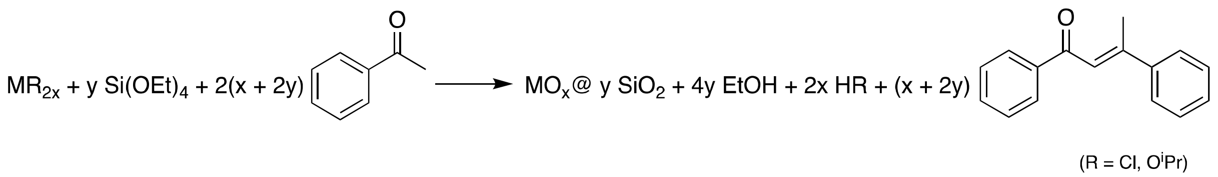

Figure S4. General reaction scheme of the synthesis of metal oxide-silica composites in acetophenone.

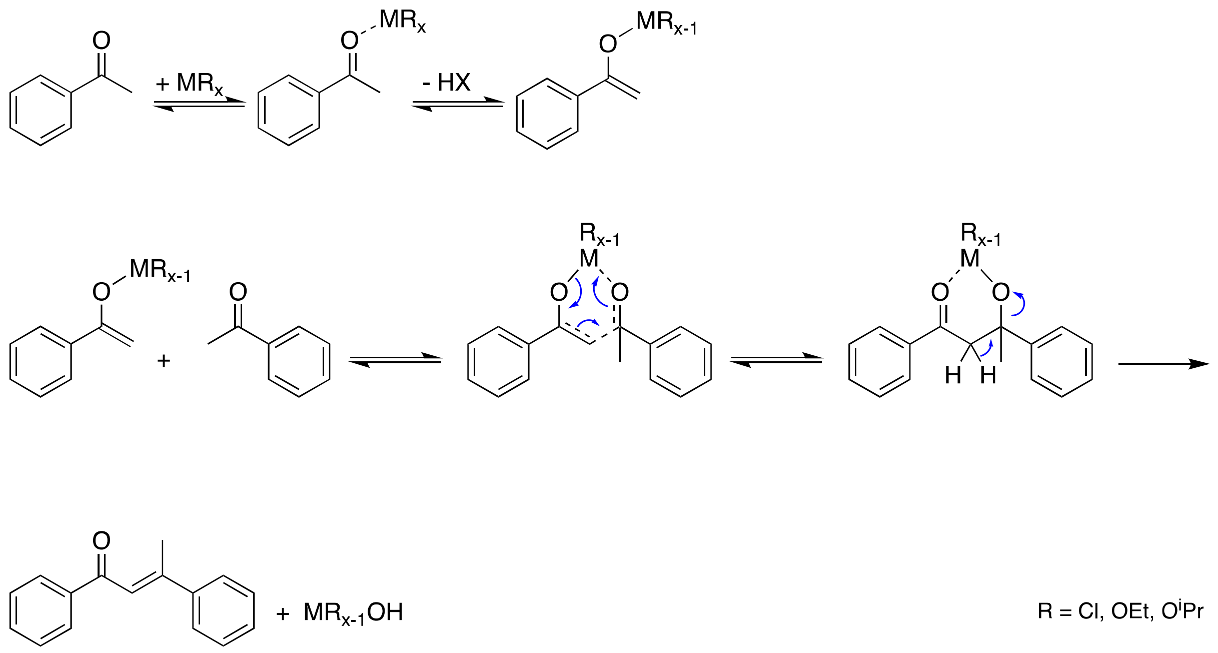

Figure S5. Proposed general reaction mechanism for the hydroxylation of the metal oxide precursor (chloride, alkoxide) via aldol-condensation of acetophenone and formation of dypnone. Lewis acidic properties of the metal oxide precursor are required to initiate the aldol-condensation. Metal oxide and silica species are grown through condensation of the hydroxylated species.

Table S1. Composition of the mesoporous silica-metal oxide materials.

**Sample**	**Nominal M (at. %)**	**Si (at. %)[b]**	**M (at. %)[b]**
**SiSn5**	10	95.0	5.0
**SiTi4**	10	96.1	3.9
**SiW6**	10	93.8	6.2
**SiMo5**	10	95.1	4.9
**SiMo6Ta8**	12.5 (Mo) + 12.5 (Ta)	85.9	6.0 (Mo) + 8.1 (Ta)
**SiNb7**	10	92.8	7.2
**SiNb20**	25	79.9	20.1
**SiNb42**	50	57.1	42.9
**SiNb65**	75	34.5	65.5
**SiNb82**	90	17.5	82.5

[a] percentage of metal added to the synthesis; [b] determined by EDS analysis.

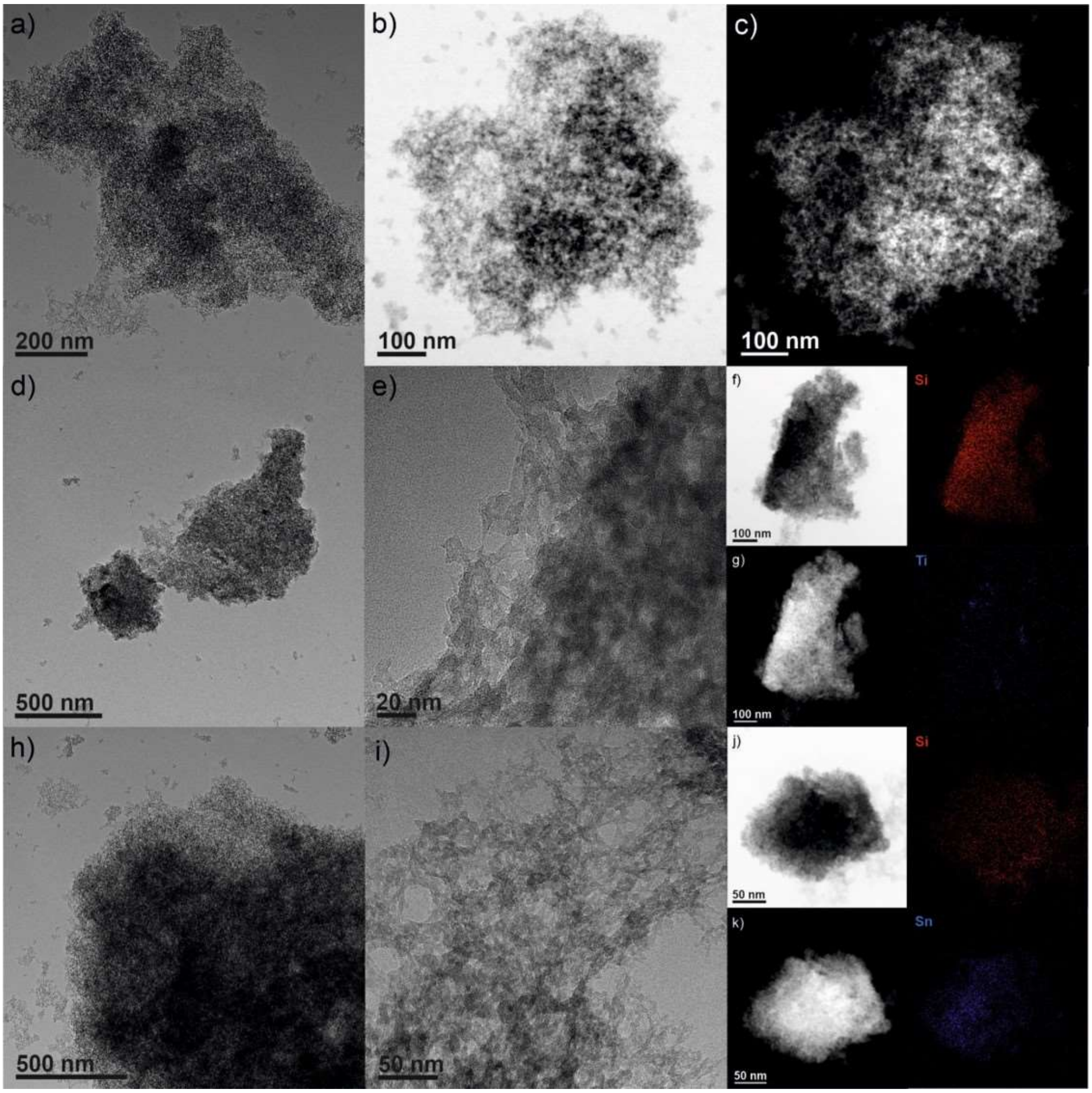

Figure S6. TEM and HAADF-STEM images of a,b,c) SiO_2, d,e, f, g) SiTi4 and h,i, j, k) SiSn5, and corresponding elemental maps.

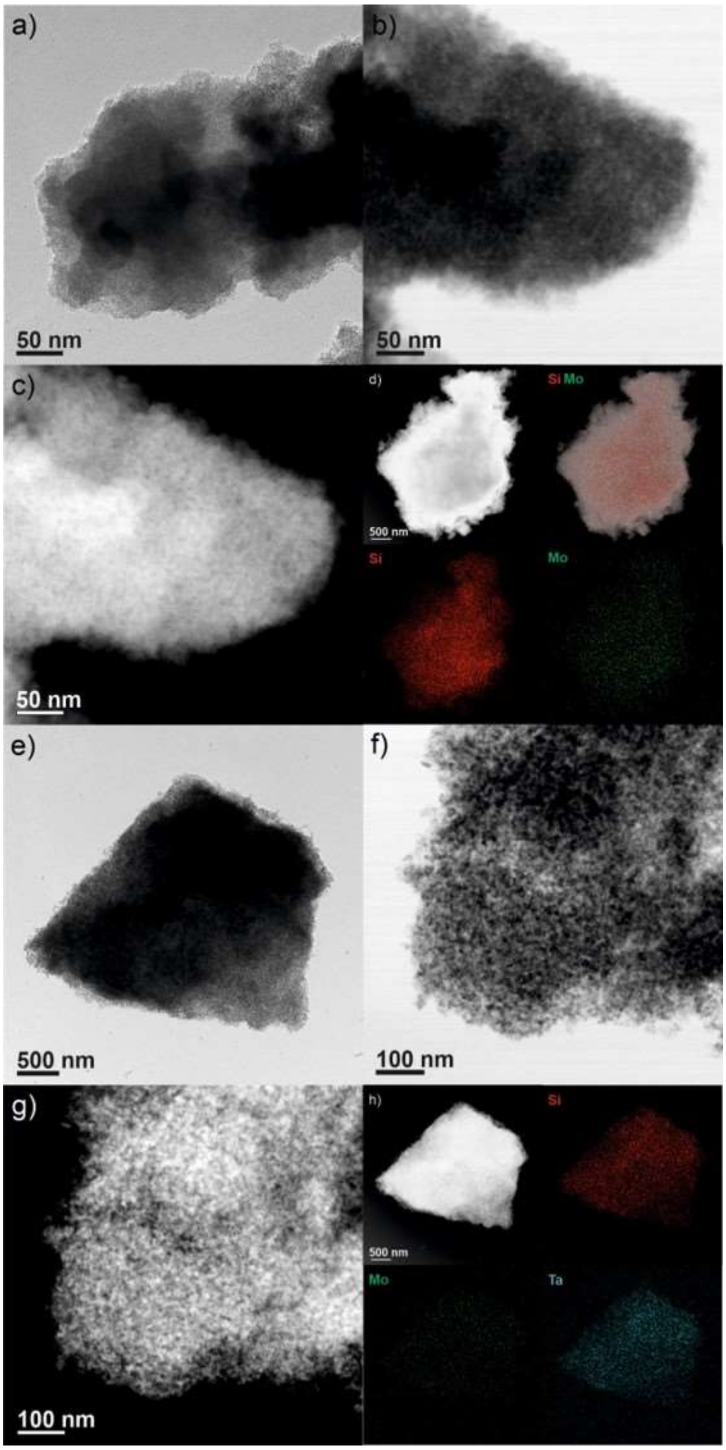

Figure S7. TEM and HAADF-STEM images of a,b,c,d) SiMo5 and e,f, g, h) SiMo6Ta8, and corresponding elemental maps.

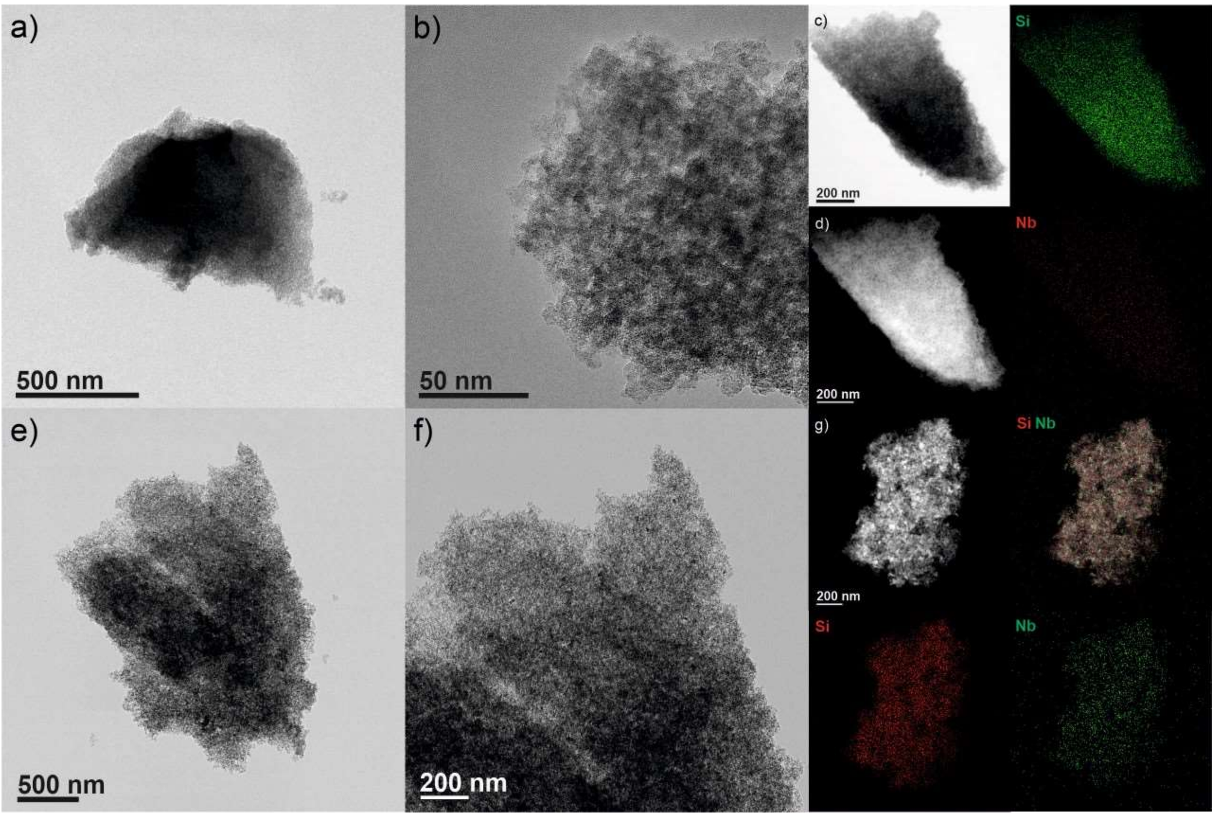

Figure S8. TEM and HAADF-STEM images of a,b,c,d) SiNb7 and e,f,g) SiNb20, and corresponding elemental maps.

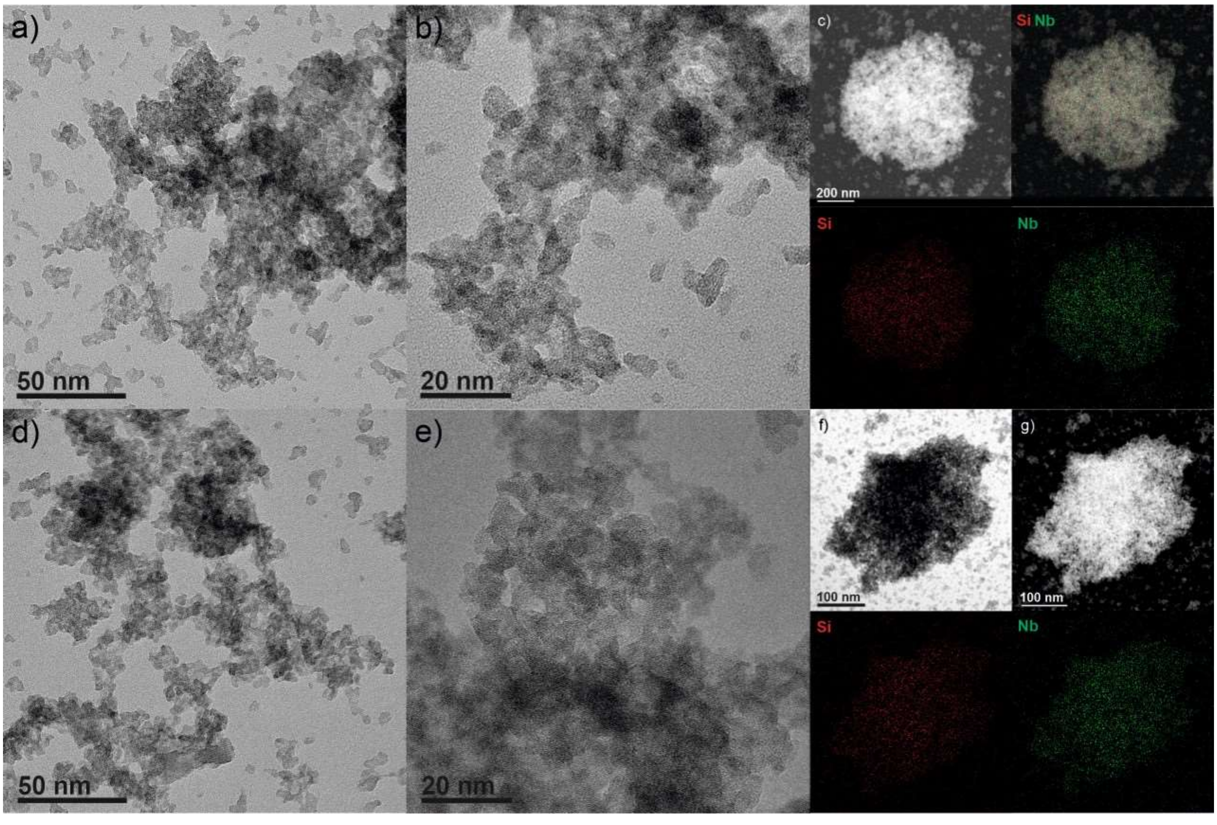

Figure S9. TEM and HAADF-STEM images of a,b,c) SiNb65 and d,e,f,g) SiNb82, and corresponding elemental maps.

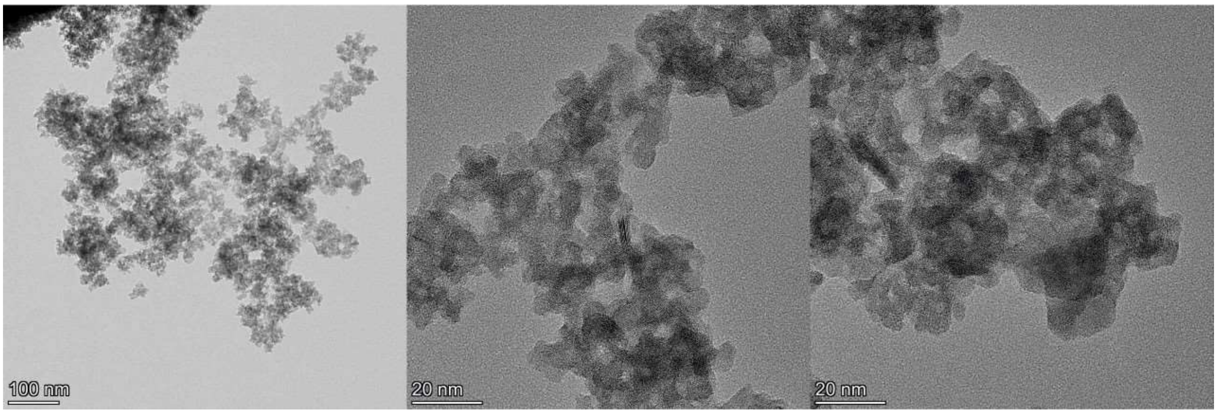

Figure S10. TEM images of Nb_2O_5 NPs calcined at 400 °C under air.

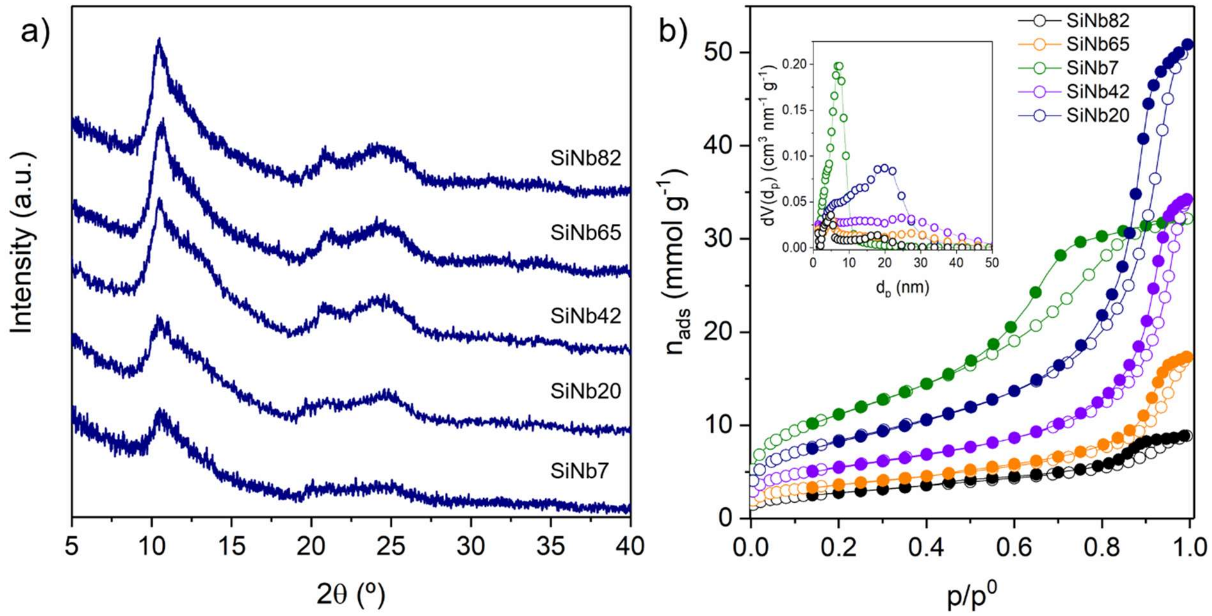

Figure S11. a) XRD patterns and b) N_2 adsorption isotherms at −196 °C of SiNbx materials (inset in b shows the pore size distributions).

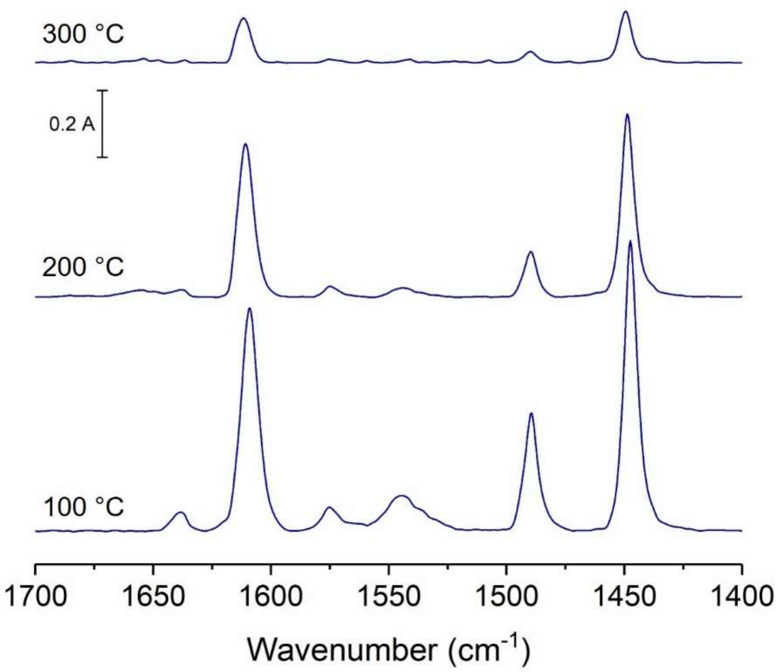

Figure S12. FT-IR spectra of pyridine adsorbed on SiNb42 at 100 °C, 200 °C, and 300 °C.

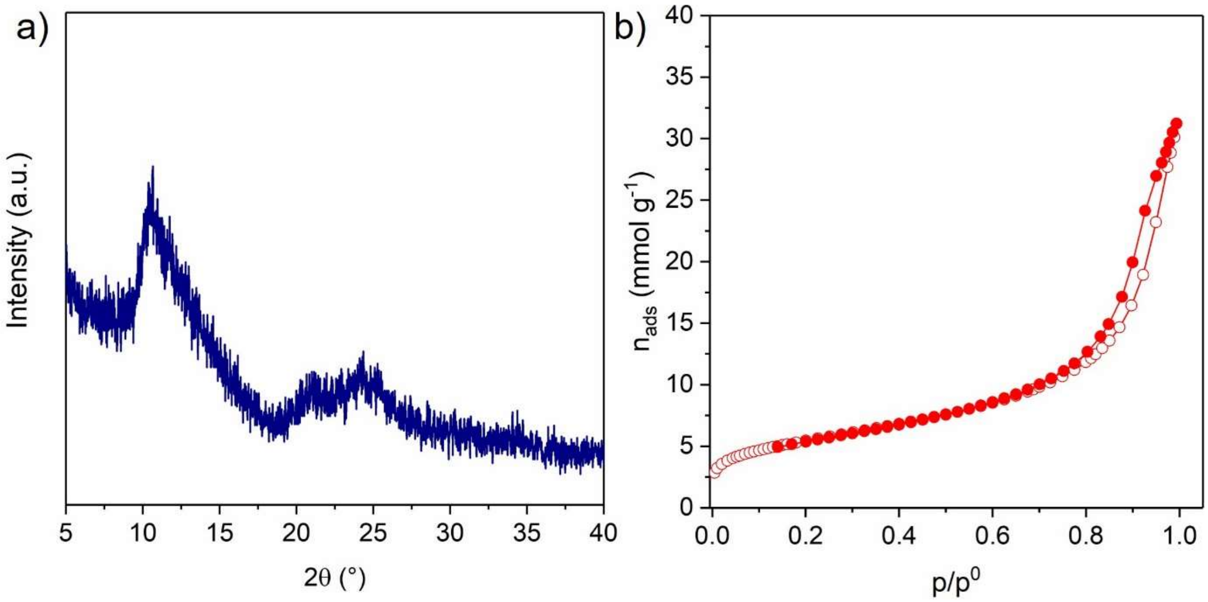

Figure S13. a) XRD pattern and c) N_2 adsorption isotherm at -196 °C of SiNb42 after catalytic tests and thermal regeneration treatments at 400 °C under air.

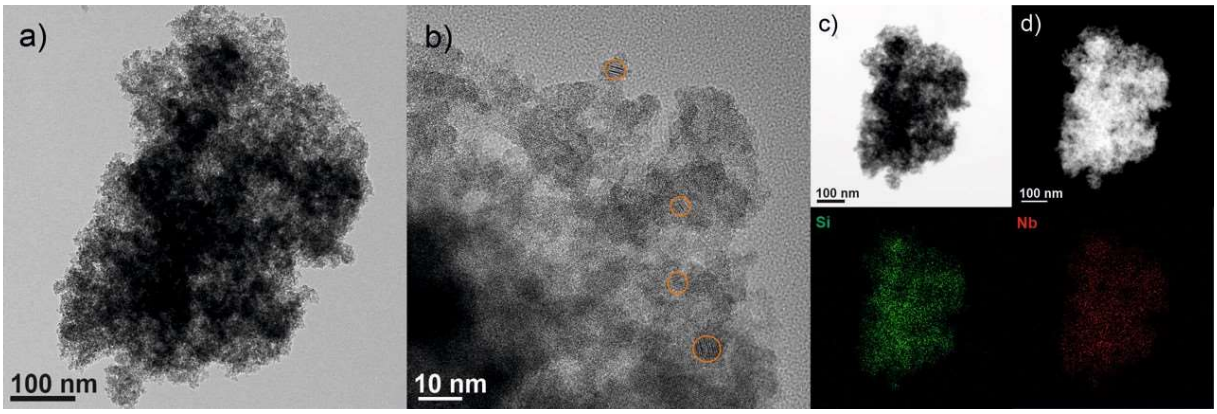

Figure S14. TEM images of SiNb42 after catalysis and thermal treatments at 400 °C under air.

Table S2. Catalytic results for SiNb42 and literature data for fully inorganic silica-based catalysts, tested for the Fur/acetone condensation.

	**Catalyst**	**Acet:Fur (mol)** [a]	**T (°C)** [a]	**t (h)** [a]	**Cat:Fur (m/m)**	**Conv (%)** [a]	**Product yield (%)**		**Ref**
							**C8**	**C13**	
1	SiNb42	7.6	140	5	0.20	75	62	8	This work
2	$20K_2O$/12wt%MgAl-SBA-15 [b]	5.0	50	3	0.08	83	19	19	1
3	Zr_{25}/m-SiO_2 [c]	10	140	2	0.42	100	76	13	2
4	SiO_2@MgAl core shell LDH [d]	10	50	4	0.16	28	7	1	3
5	Cu/Al-MCM-41	40	175	24		98	92		4

[a] Acet:Fur = mole ratio of acetone:Fur, T = reaction temperature, t = reaction time, Conv. = Fur conversion. [b] For this catalyst the activity decreased in consecutive runs. [c] Zr_{25}/m-SiO_2 = zirconium (25 wt%) on mesoporous silica. [d] LDH= layered double hydroxide.

References

1. Arumugam, M.; Kikhtyanin, O.; Osatiashtiani, A.; Kyselová, V.; Fila, V.; Paterova, I.; Wong, K. L.; Kubička, D. Potassium-Modified Bifunctional MgAl-SBA-15 for Aldol Condensation of Furfural and Acetone. *Sustain. Energy Fuels* **2023**, *7* (13), 3047–3059.

2. Balaga, R.; Yan, P.; Ramineni, K.; Du, H.; Xia, Z.; Marri, M. R.; Zhang, Z. C. The Role and Performance of Isolated Zirconia Sites on Mesoporous Silica for Aldol Condensation of Furfural with Acetone. *Appl. Catal. A Gen.* **2022**, *648*, 118901.

3. Kondratowicz, T.; Slang, S.; Dubnová, L.; Kikhtyanin, O.; Belina, P. .; Capek, L. Controlled Silica Core Removal from SiO2@MgAl Core-Shell System as a Tool to Prepare Well-Oriented and Highly Active Catalysts. *Appl. Clay Sci.* **2022**, *216*, 106365.

4. Gandhi, P.; Saha, B.; Vedachalam, S.; Dalai, A. K. Renewable Fuel Intermediates from Furfural over Copper-Loaded Mesoporous Aldol Condensation Catalysts. *Sustain. Energy Fuels* **2023**, No. 17, 4260–4272.
